# Enteral Administration of a Methylene Blue and 20% Lipid Emulsion Slurry to Aid in Diagnosis of Intraoperative Thoracic Duct Injury

**DOI:** 10.1016/j.atssr.2025.06.016

**Published:** 2025-07-21

**Authors:** Ali Hemyari, Allison B. Davila, Michael A. Evans

**Affiliations:** 1 Division of Pediatric Anesthesiology, Department of Anesthesiology, Northwestern University Feinberg School of Medicine, Chicago, Illinois; 2 Division of Pediatric Surgery, Department of Surgery, Northwestern University Feinberg School of Medicine, Chicago, Illinois

## Abstract

Thoracic duct identification during surgery in small children is often difficult or impossible secondary to patient size, surgical approach, or pathologic anatomy. Intraoperative detection of injury to the thoracic duct or other lymphatics is even more challenging, especially if the patient’s anatomy is nonstandard. We describe a congenital heart disease patient with a preexisting pleural effusion that was administered an enteral slurry of methylene blue and 20% lipid emulsion intraoperatively to help delineate anatomy and detect chyle leakage. The slurry allowed for lymphatics visualization, but not thoracic duct identification.

Postoperative chylous effusions are a common complication in pediatric cardiac surgery, both after sternotomy and thoracotomy, with a reported incidence of 2.8%.^1^ A postsurgical chylothorax may form as a result of any number of insults, including direct damage to the thoracic duct, injury to accessory lymphatic vessels during surgical dissection, elevations in central venous pressure from single-ventricle palliative surgery, or central venous thrombosis. Diagnosis of chylous effusion is confirmed when pleural fluid triglyceride level is measured as >110 mg/dL. Herein, we present the enteral use of a methylene blue and 20% lipid emulsion slurry to delineate anatomy intraoperatively when chylous effusion was suspected. Written informed consent for publication of the present case was obtained from the patient’s guardian, and this article adheres to the applicable Enhancing the QUAlity and Transparency of health Research (EQUATOR) guidelines.

An 11-month-old, 9.8 kg girl with right aortic arch, aberrant left subclavian artery, left-sided ligamentum arteriosum, and subsequent esophageal compression was brought to the operating theatre for vascular ring repair. Anesthesia was induced, peripheral intravenous and arterial lines placed, and the patient intubated with a tube capable of recurrent laryngeal nerve monitoring. The patient was placed into the right lateral position, and a left paravertebral block was performed. Upon initial thoracotomy, a large pleural effusion was drained, which was not visualized on preoperative chest film. Fluid continued to well up in the left hemithorax throughout dissection. Due to the patient’s size, surgical exposure, and an enlarged thymus that distorted anatomy, the thoracic duct could not be identified. Given the ongoing fluid accumulation and absence of an identifiable thoracic duct, the cardiac surgeon requested a fat challenge by way of enteral administration of a methylene blue and lipid slurry.

The slurry was created using 1.5 mg/kg methylene blue and 30 mL 20% lipid solution (Figure 1) and administered enterally via orogastric tube. Sixty-four minutes after administration, the surgeon visualized blue-stained lymphatics on the lung’s surface. At this time, no chylous nor blue-tinged fluid was visualized in the chest. Surgery continued, and ultimately included esophageal mobilization, ligation and division of the ligamentum arteriosum, ligation and division of Kommerell diverticulum, and reimplantation of the aberrant left subclavian artery into the left common carotid artery. At time of closure, there was no evidence of fluid accumulation in the chest, and there was no observed change of fluid character over time: all visualized methylene-blue-stained fluid during the operation was contained to the intact lymphatic network on the surface of the lung. Given effusion presence at the start of surgery, the surgeon left a 16F thoracostomy tube in place. After closure and Valsalva maneuvers, there was no pleural fluid—serous, milky, bloody, or blue—visualized in the chest tube.

**Figure 1:**
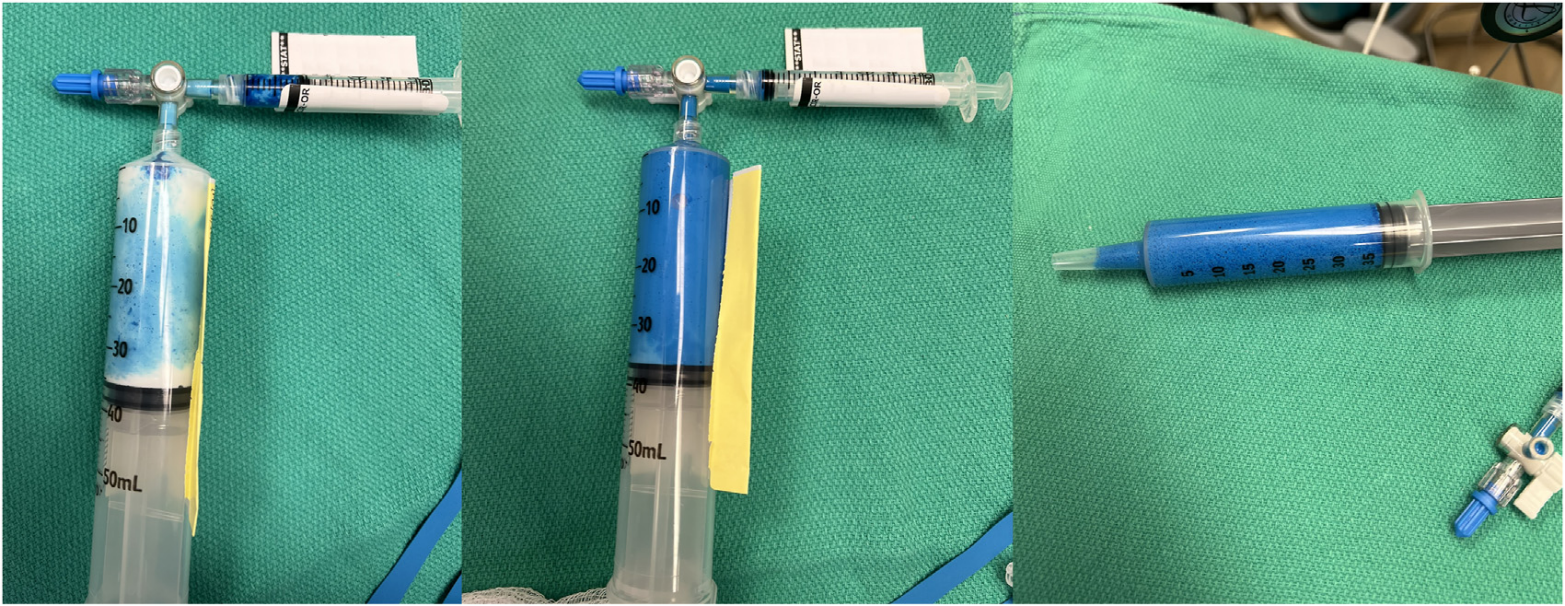
Methylene blue slurry. The 20% lipid emulsion and methylene blue combination utilizing a 3-way-stopcock to form a slurry. The slurry was transferred to a slip-tip syringe for administration by orogastric tube.

The patient was extubated and taken to the floor on no inotropic support. Unfortunately, the patient’s chest tube output became frankly chylous after their first meal, 6 hours postoperatively. A chylous effusion with a triglyceride content of 266 mg/dL was confirmed via fluid analysis. Enfaport formula (Mead Johnson & Co) was initiated as part of a low-fat diet, the thoracostomy tube removed on postoperative day 1 due to low output, and the patient discharged home on postoperative day 2. The patient’s chest x-ray film was clear at her follow-up visit on postoperative day 6.

## Comment

Although enteral administration of a methylene blue and 20% lipid solution slurry did not reveal the patient’s thoracic duct or a chyle leak intraoperatively, it did opacify their lymphatics, demonstrating the technique’s potential utility to highlight the thoracic duct or source of lymphatic leak in pediatric cases with nonstandard or pathologic anatomy. It is unclear why this patient presented with a large pleural effusion preoperatively, but it correlates with effusion resolution with only dietary modification. A serious surgical lymphatic or duct injury would not allow for a postoperative day 2 discharge, nor have presented with a low volume postoperative effusion. Regarding intraoperative observation of lymphatic opacification with milky-blue lymph, understanding the underlying pharmacokinetics of enteral methylene blue and lipids along with gastric uptake time are paramount for accurate clinical decision-making.

Ultimately, the thoracic duct could not be visualized intraoperatively. Several methods have been utilized previously to opacify the thoracic duct in adults, including ice cream,^2^ heavy cream,^3^ and olive oil.^4^ In patients fasted for surgery, lymphatic vessels collapse, and lymph is transparent. This transparency makes differentiation from surrounding tissues nearly impossible, especially in pediatric patients. Enteral lipid administration allows long-chain fatty acids to be chylomicronized in the small intestine, dramatically increasing lymphatic flow. In the case of our pediatric patient, the addition of methylene blue to the slurry augmented visualization of tiny, translucent structures. A high-volume effusion would also be augmented more than the low-volume effusion seen in our patient.

**Figure 2:**
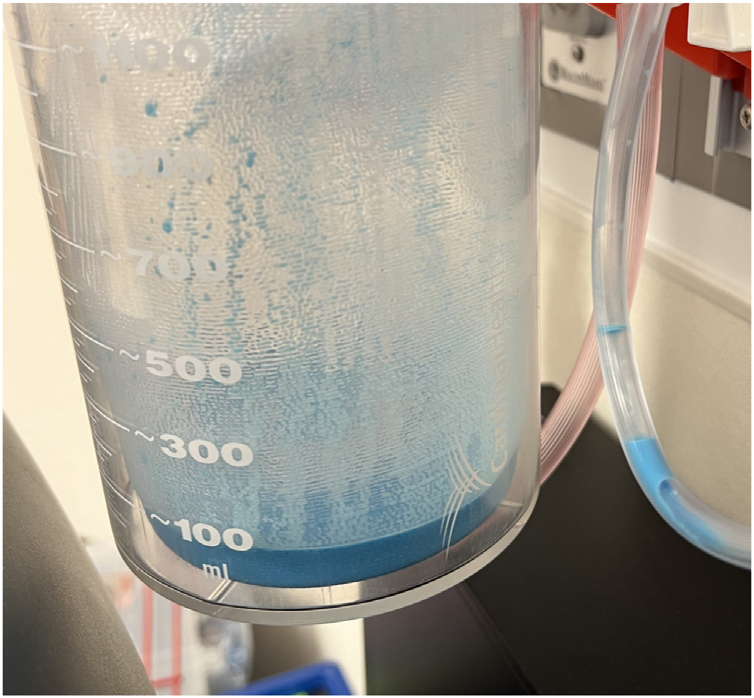
Gastric residual volume. Despite lymphatic visualization after adequate lipid and methylene blue uptake, a significant volume of residual slurry was suctioned from the patient’s stomach prior to extubation.

Methylene blue can also be directly injected into lymph nodes to determine lymphatic leak location, but this method requires visible lymph nodes or a thoracic duct that is large enough to inject, neither of which were possible in this patient. Fortunately, enteral methylene blue is rapidly absorbed, and can be detected in urine less than one hour after oral administration.^5^ This rapid uptake suggests that the rate-limiting step for the technique utilized in this patient was fat absorption. It is possible that earlier administration may have helped delineate thoracic duct anatomy or even yielded chylous effusion diagnosis intraoperatively. Thankfully, given rapid visualization of methylene blue, there was intraoperative reassurance that there was no large duct leak requiring intervention. In contrast, in adults given postinduction enteral olive oil, the median duration of time to thoracic duct identification was 100 minutes.^4^ Preoperative lipid administration may therefore increase the timeliness of thoracic duct opacification, but mandating rapid sequence inductions in all vascular ring patients and risking aspiration is prohibitive. In fact, a notable volume of slurry was suctioned from the patient’s stomach prior to extubation (Figure 2), which is a good reminder that gastric transit times should not be forgotten. Interestingly, despite the chylous effusion, no blue fluid was drained by the thoracostomy tube. This finding may indicate the necessity of coadministering lipids and methylene blue. Given the benefits of simple oral lipid administration that have already been demonstrated in adults,^4^^,^^5^ the early, postinduction, protocolized prophylactic administration of enteral methylene blue and lipids is a safer method to aid in surgical landmark visualization. For this reason, an institutional protocol has been developed implementing enteral administration of slurry immediately postinduction, allowing maximal uptake time for the lipid component, with the goal of consistent thoracic duct identification even in challenging cases.
